# Public transit infrastructure and heat perceptions in hot and dry climates

**DOI:** 10.1007/s00484-021-02074-4

**Published:** 2021-01-26

**Authors:** Yuliya Dzyuban, David M. Hondula, Paul J. Coseo, Charles L. Redman

**Affiliations:** 1 Office of Core Curriculum, Singapore Management University, Singapore, Singapore; 2 School of Geographical Sciences and Urban Planning, Arizona State University, Tempe, AZ USA; 3 The Global Institute of Sustainability and Innovation, The Julie Ann Wrigley Global Futures Laboratory, Arizona State University, Tempe, AZ USA; 4 The Design School, Herberger Institute for Design and the Arts, Arizona State University, Tempe, AZ USA; 5 School of Human Evolution and Social Change, College of Liberal Arts and Sciences, Arizona State University, Tempe, AZ USA

**Keywords:** Urban climate, Urban design, Public transit infrastructure, Outdoor thermal comfort, Heat perception, Extreme heat, Human biometeorlogy

## Abstract

Many cities aim to progress toward their sustainability and public health goals by increasing use of their public transit systems. However, without adequate protective infrastructure that provides thermally comfortable conditions for public transit riders, it can be challenging to reach these goals in hot climates. We took micrometeorological measurements and surveyed riders about their perceptions of heat and heat-coping behaviors at bus stops with a variety of design attributes in Phoenix, AZ, USA, during the summer of 2018. We identified the design attributes and coping behaviors that made riders feel cooler. We observed that current infrastructure standards and material choices for bus stops in Phoenix are insufficient to provide thermal comfort, and can even expose riders to health risks. Almost half of the study participants felt hot or very hot at the time they were surveyed, and more than half reported feeling thermally uncomfortable. On average, shade reduced the physiological equivalent temperature (PET) by 19 °C. Moreover, we found significant diurnal differences in PET reductions from the shade provided by various design attributes. For instance, all design attributes were effective in reducing PET in the morning; however, a vegetated awning did not provide statistically significant shade reductions in the afternoon. Temperatures of sun-exposed surfaces of man-made materials exceeded skin burn thresholds in the afternoon, but shade was effective in bringing the same surfaces to safe levels. Aesthetically pleasing stops were rated as cooler than stops rated as less beautiful. We conclude that cities striving to increase public transit use should prioritize thermal comfort when designing public transit stops in hot climates.

**Supplementary Information:**

The online version contains supplementary material available at 10.1007/s00484-021-02074-4.

## Introduction

Episodes of extreme heat are expanding in intensity, duration, and scope in many global cities. At the same time, many cities are changing zoning codes away from those that are car-oriented to those that are walking- and transit-oriented to reduce greenhouse gas emissions and achieve other sustainability goals (Ewing et al. 2008). Yet, in many cases, pedestrian and transit-oriented infrastructure has not been upgraded with pedestrian thermal comfort in mind, potentially creating dangerous exposure to heat. Growth in transit infrastructure will likely increase the number of people exposed at the same time that uncomfortable, or even intolerable, heat is becoming more frequent and severe. The intersection of these two trends—increasing pedestrian exposure to extreme heat and investment in additional pedestrian and transit-oriented infrastructure—has not been adequately addressed by research.

Weather is known to influence the use of bus and rail-oriented public transit systems (e.g., Kalkstein et al. 2009; Kuby et al. 2004; Li et al. 2011; Singhal et al. 2014; Stover and McCormack 2012). For instance, a study in Brisbane, Australia, found that rainfall was associated with increased ridership at areas with more shelters while ridership decreased in areas with less rain protection. In the same study, wind increased ridership in remote locations, possibly due to a cooling effect in a subtropical climate; and high humidity was associated with reduction in ridership across the system (Tao et al. 2016). Rain and low temperatures are frequently cited as deterrents to transit ridership elsewhere (Singhal et al. 2014), but literature that would offer insights into riders’ challenges during high heat incidents is scarce. Fraser and Chester (2016) assessed public transit riders’ length of exposure to heat while walking and waiting for bus service on a regional scale for Los Angeles County, CA, and Maricopa County, AZ. The authors noted that the extent to which transit shelters and small-scale cooling amenities can protect from extreme heat exposure is not well understood and that more research is needed to assess whether current shelter designs are effective in mitigating heat-related health risks (Fraser and Chester 2016). Measuring exposure to surface and air temperatures in actively used urban areas is essential for understanding the health effects on users and for successful adaptation for future urban warming and changes in urban planning and design (Vanos et al. 2016).

Moreover, bus stops, as well as other types of urban furniture, can provide aesthetic and symbolic qualities in addition to their primary functionality to offer services that are more attractive to users. A study in Brazil identified several attributes that are associated with pleasantness at bus stops, such as the availability of seating, the presence of vegetation, curved shelter structures, and a back wall (Pizzato and Guimarães 2012). In hot climates, pleasantness can also be related to alliesthesia, the perception of external stimuli that provides cooling as pleasant (Heng and Chow 2019; Johansson et al. 2018). Thus, a bus stop that provides shade (and potentially other cooling amenities) can be perceived as pleasant in hot, sun-exposed locations.

This research aims to quantify the environmental and social impact of various design attributes found at bus stops using both microclimate and surface temperature measurements and subjective assessments of heat and pleasantness, and to document the main behavioral cooling strategies of riders during the hottest summer months. Our study objectives are to (1) assess how microclimate conditions at bus stops are affected by shade from available design attributes and diurnal changes; (2) investigate surface temperature variability of prevailing materials, the impact of shade on surface temperatures, and diurnal differences in surface temperatures in relation to the risks to human health; (3) analyze perceptions of heat, pleasantness, and cooling benefits provided by available design attributes; and (4) document riders’ heat adaptation behaviors while walking and waiting at bus stops.

## Methodology

### Study site

**Fig. 1:**
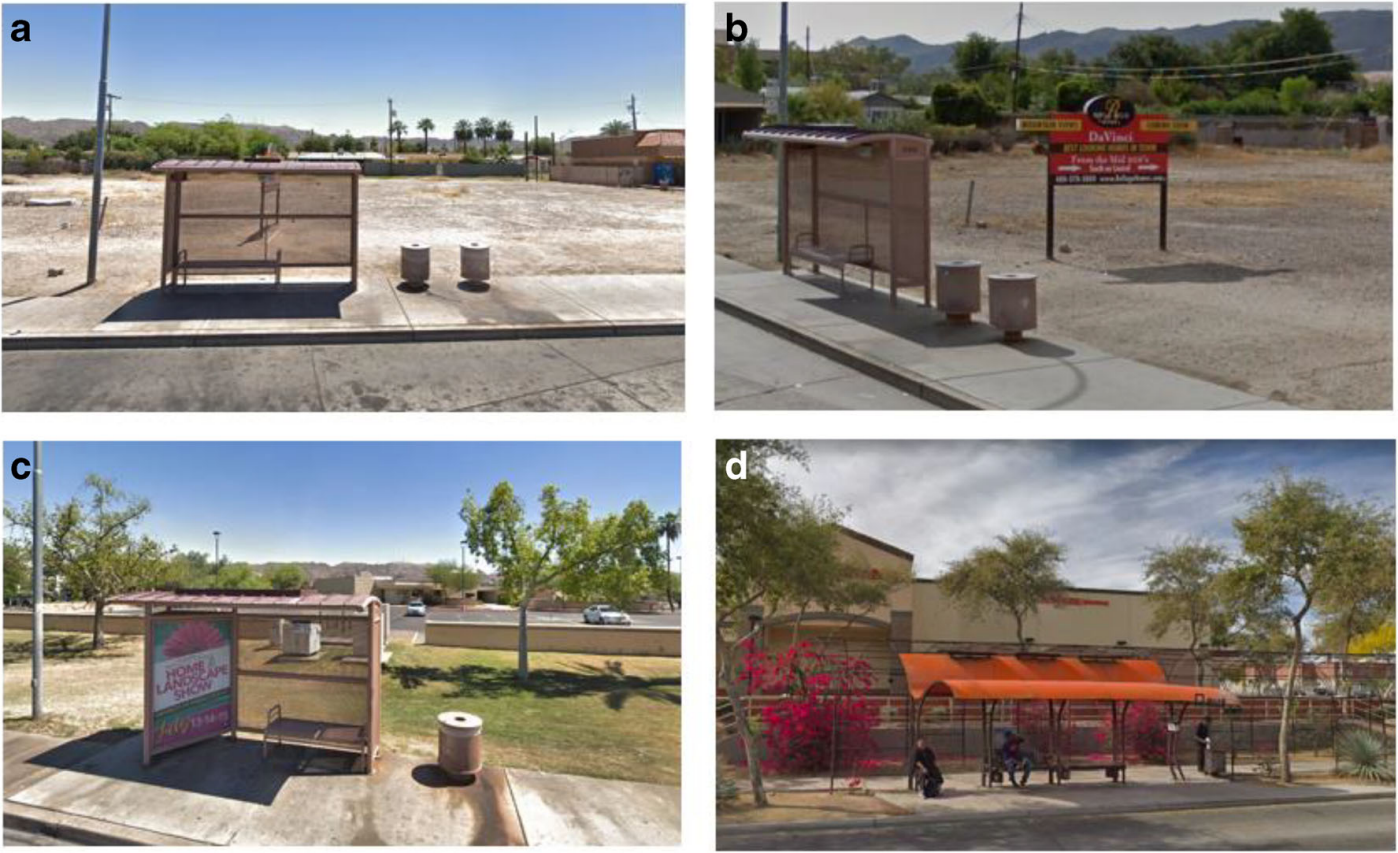
Types of bus stops and design attributes examined during the field campaign between June 6 and July 27, 2018. **a** Standard bus stop shelter. **b** Standard bus stop shelter with a standalone advertising sign used for shade protection by bus riders. **c** Standard bus stop shelter with integrated advertising panel and minimal landscaping. **d** Enhanced bus stop shelter with integrated artwork, trees, landscaping, and vegetated metal trellis

This study is set in the City of Phoenix, AZ, the fifth largest by population in the USA. Phoenix is located in the Sonoran desert (33.4484° N, 112.0740° W, 331 m above sea level) and is one of the hottest cities in the USA experiencing about 110 days each year during which maximum daily temperatures exceed 38 °C (National Weather Service - NWS Phoenix n.d.). Its climate is characterized as hot arid desert (Köppen-Geiger BWh) (Kottek et al. 2006). Field measurements were conducted in South Mountain Village, a predominantly Latinx neighborhood, where poverty rates exceed 40% in some census tracts (Bolin et al. 2005). Residents have low car ownership compared to the rest of the city and rely on public transit. In addition to socio-economic vulnerability, residents of the neighborhood have high exposure to heat due to a lack of vegetation, a high concentration of impervious surfaces, and limited air conditioning in residents’ houses (Harlan et al. 2006, 2013). We selected bus stops in South Mountain Village (Fig. 1) based on variability in the design attributes, such as differences in shelter design, the presence of advertising, vegetation, differences in seating options/configuration, and average daily ridership (Valley Metro Bus Ridership | Valley Metro GeoCenter n.d.). Even though bus stops without any seating or shade structures are also present in the region, they were not included in the study due to very low ridership at such stops.

Standard bus stop shelters (Fig. 1a, b, and c) are the most common and consist of a painted metal shelter with a curved solid canopy, perforated back and side walls with or without an advertising panel, and a perforated metal bench. In addition, standalone metal advertising signs are sometimes present in the vicinity of the bus stop (Fig. 1b), which riders occasionally use for shade cover, and thus measurements near them were also included. Some standard bus stop shelters have minimal landscaping with sparse trees as shown in Fig. 1c. Enhanced bus stops with integrated artwork and landscaping (Fig. 1d) are examples of collaboration between the City of Phoenix and local artists. Only a few such shelters exist throughout the city; they consist of a polycarbonate canopy with art elements, several metal benches, and individual seating. An entwined metal trellis forms a vegetated awning behind the stop. However, vine density is not consistent across the trellis structure; patches with sparse or no vegetation are common due to maintenance and irrigation challenges. Trees and shrubs have been planted around the art stops but are rarely present at others.

Data were collected along a major arterial road. Four bus stops with standard shelter designs and two enhanced bus stops were selected, all facing north to control for the differences in sun position and shade patterns. Selected bus stops incorporated a variety of design attributes such as a metal or polycarbonate canopy, standalone vertical metal signs, trees, and a metal trellis with vines. The average daily ridership at the selected stops ranged from 29 riders per day at minor intersections, to 107 riders per day at major intersections (Valley Metro Bus Ridership | Valley Metro GeoCenter n.d.). These numbers are near the average for the region: the average daily ridership in Phoenix per bus stop between April 2016 and June 2018 was 24 riders with a standard deviation of 41^1^ (Valley Metro Bus Ridership | Valley Metro GeoCenter n.d.). Thus, we estimate that the current sample is a reasonable representation of the ridership in residential areas of Phoenix.

### Study design and data collection

#### Meteorological measurements

Measurements were taken on 19 days between June 6 and July 27, 2018, with clear skies and maximum daily ambient temperatures in the range 38–43 °C. Data were collected three times daily in 2-h intervals: 7:00–9:00 a.m., noon–2:00 p.m., and 3:00–5:00 p.m. These times were selected because they are the hours of peak ridership. We recorded environmental conditions at each stop in sun-exposed locations as well as in shaded locations provided by design attributes of the stops (Table 1).

**Table 1:** Description of design attributes, average daily ridership (provided by Valley Metro), and surveys collected during the field campaign between June 6 and July 27, 2018

Stop no.	Collected surveys	Average daily ridership	Bus stop shelter description	Tree	Vegetated awning	Standalone advertising sign
1	9	29	Painted metal	No	No	No
2	24	70	Painted metal	No	No	Yes
3	6	30	Painted metal with integrated advertising panel	Yes	No	No
4	9	35	Painted metal with integrated advertising panel	No	No	Yes
5	18	36	Painted metal structure with polycarbonate canopy	Yes	Yes	No
6	17	102	Painted metal structure with polycarbonate canopy	Yes	Yes	No

We used Kestrel 4400 Heat Stress Meters to measure ambient temperature, globe temperature, relative humidity, and wind speed at each stop. These sensors were attached to tripods at a height of 1.1 m, which is the center of gravity of a standing human (Middel et al. 2016). Surface temperatures were taken with Extech IR260 infrared thermometers. All instruments complied with ISO 7726 standards for sensor measurement range and accuracy (ISO 7726 2001). Mean radiant temperature (Tmrt) was calculated from observed measurements according to the equation:

$$ Tmrt={\left[{\left( Tg+273\right)}^4+\frac{1.1\times {10}^8{Va}^{0.6}}{\varepsilon\ {D}^{0.4}}\times \left( Tg- Ta\right)\right]}^{1/4}-273 $$

with Ta = ambient temperature [°C]; Tg = globe temperature [°C]; Va = wind speed [ms^−1^]; D = globe diameter [m]; ε = globe emissivity (ISO 7726 2001).

Tmrt and measured microclimate parameters were used to calculate the physiological equivalent temperature (PET). PET, which is widely used in thermal comfort studies, defines a condition at which the human body is at heat balance indoors compared to the outdoor conditions (Mayer and Höppe 1987). The effect of various design attributes on PET was explored using factorial analysis of variance (factorial ANCOVA) (Warner 2013, p. 501), with ambient temperature from the local airport station as a covariate and sun and shade values from available design attributes as factors. Differences in PET between sun and shade for each design attribute were averaged and visualized with boxplots.

#### Surface temperatures

Surface temperatures of prevalent materials available at bus stops were measured in the sun and in the shade where such conditions were available. Measurements were taken three times during each shift at equal intervals. We calculated various descriptive statistics, including the percent of observations above thresholds for 1-min and 5-s skin burns (ISO 13732-3 2010), and mean differences between the same material types in the sun and shade. Statistical significance of differences between sun and shade exposed materials was explored with factorial ANCOVA, using ambient temperature from the local airport station as a covariate and material types as factors.

#### Field surveys

Bus riders waiting at the six study stops were surveyed during the same time intervals during which meteorological measurements were taken. Requests to participate in the surveys were rarely declined. The survey took about 5 min to administer and consisted entirely of closed ended questions. Participants were offered cold water in appreciation of their time and effort. After each survey was completed, survey administrators noted the respondent’s apparent gender, sun exposure, and meteorological conditions at the stop (ambient temperature, globe temperature, wind speed, and relative humidity).

The survey (Supplementary material) consisted of three parts. The first part asked riders how they typically traveled to the bus stop and how long it took them to get to there, how long they typically waited for the bus, what they did while waiting, and what their strategies were for coping with heat while waiting. The second part included questions about perception of the bus stop infrastructure and thermal comfort (following Knez et al. 2009). We asked about green and gray infrastructure elements that riders might perceive to have cooling benefits. The last part included questions about riders’ primary transit mode and vehicle ownership, the reason for the bus trip, income, and age. This project was approved by the Institutional Review Board of Arizona State University (Study #00006309).

We calculated Spearman’s rank-order correlation to identify relationships between responses to the survey questions and meteorological variables. Significant relationships (*p* < 0.05) were further explored with linear regression models.

## Results

### Meteorological measurements

We recorded 241 microclimate measurements in sun and shade conditions at bus stops (Table 2). Across all stops, the mean PET was 36.1 °C in the shade and 53.4 °C in the sun in the morning and 49.5 °C in the shade and 68.2 °C in the sun in the afternoon. The maximum PET we observed was 81.6 °C, recorded at 12:20 pm on the 26th of July, 2018, in the sun at the bus stop with a standard shelter type and no vegetation.

**Table 2:** Mean and standard deviation (sd) of microclimate variables in the morning (a) and afternoon (b) collected during the field campaign between June 6 and July 27, 2018 (*N* = 241)

Variable	Ambient temperature [°C]	Globe temperature [°C]	Wind speed [ms^−1^]	Relative humidity [%]	PET [°C]
(a) Morning					
All locations mean (sd)	34 (2.5)	38.9 (4.9)	0.8 (0.6)	26.5 (13.5)	44.1 (10)
Sun locations mean (sd)	34.6 (2.4)	43.3 (2.7)	1 (0.7)	27 (13.9)	53.4 (6.3)
Shade locations mean (sd)	33.6 (2.5)	35.1 (2.8)	0.7 (0.5)	26 (13.2)	36.1 (3.8)
Sun-Shade difference	1	8.2	0.3	1	17.3
(b) Afternoon					
All locations mean (sd)	41.5 (2)	48.1 (4.5)	1.2 (0.8)	14.2 (9)	58.6 (11.3)
Sun locations mean (sd)	41.8 (2.1)	52 (2.1)	1.5 (0.9)	14.6 (9.2)	68.2 (7.4)
Shade locations mean (sd)	41.2 (1.7)	44.3 (2.4)	1 (0.7)	13.7 (8.8)	49.5 (5.2)
Sun-Shade difference	0.6	7.7	0.5	0.9	18.7

Factorial ANCOVA revealed significant differences between the PET and time of day (*F* = 6.719, *p* = 0.001). Pairwise comparisons showed significant difference between 7:00–9:00 a.m., and 12:00–2:00 p.m. (mean difference = − 11.126, *p* = 0.01) and between 7:00–9:00 a.m. and 3:00–5:00 p.m. (mean difference = − 12.356, *p* = 0.02), but the difference between 12:00–2:00 p.m. and 3:00–5:00 p.m. was not significant (*p* = 0.874). Hence, 12:00–2:00 p.m. and 3:00–5:00 p.m. measurements were combined for exploring diurnal differences. Factorial ANCOVA showed that shade significantly influenced PET in the morning (*F* = 48.045, *p* < 0.001, partial eta squared = 0.772) and in the afternoon (*F* = 85.665, *p* < 0.001, partial eta squared = 0.733).

**Fig. 2:**
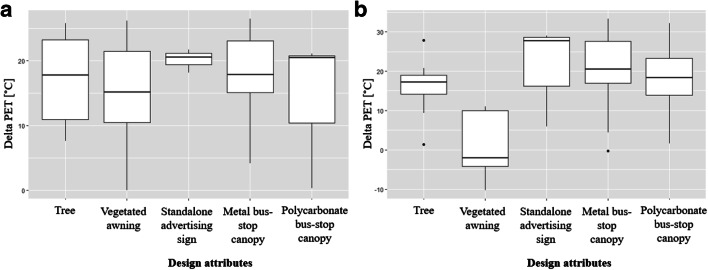
Boxplots of differences in PET between sun and shade conditions per design attribute in the morning (**a**) and afternoon (**b**) collected during the field campaign between June 6 and July 27, 2018 (*N* = 125)

In the morning, all design attributes provided shade that resulted in significantly lower PET values than those measured in the sun. The advertising sign had the largest effect on morning PET (20 °C) and the vegetated awning had the lowest (15.2 °C). In the afternoon, the shade from all design attributes provided significant PET reductions except for the vegetated awning. Differences in PET between the various shade conditions were not significant in the morning (*p* = 0.195), but they were for the afternoon (*F* = 12.581, *p* < 0.001, partial eta squared = 0.395). Pairwise comparisons showed that the vegetated awning was associated with statistically significantly smaller PET reductions than the other design attributes, making it the least effective in reducing PET. Shade from the advertising sign offered up to 20.3 °C reductions in PET on average in the afternoon; metal bus stop shelters performed slightly better than polycarbonate, 20.6 °C versus 18.2 °C, respectively (Fig. 2b).

### Surface temperatures

We recorded 1003 measurements of surface temperatures of various materials at the stops in sun-exposed and shaded conditions (Table 3). We observed large differences between sun and shade that were more pronounced in the afternoon than morning, as well as high variability in measurements for individual surfaces. Materials sampled included powder-coated metal, concrete, dirt/gravel, asphalt, and grass. Asphalt had the highest mean sun-exposed surface temperature of 54.7 °C. The single highest surface temperature measurement we recorded was for gravel/dirt, at 74.4 °C. The mean surface temperature for sun-exposed metal bench seats was 39.7 °C, with maximums above 60 °C. Grass had the lowest mean surface temperature of 38.4 °C; however, the maximum surface temperature for grass exceeded 66 °C.

**Table 3:** Mean and standard deviation (sd) of surface temperatures collected in the morning (**a**) and afternoon (**b**) during the field campaign between June 6 and July 27, 2018 (*N* = 1003); percent of values above 1-min and 5-s skin burn threshold; mean values for sun and shade and mean difference for measured materials

Type of material/object	Metal bench (powder-coated metal)	Concrete	Dirt/gravel	Asphalt	Grass
(a) Morning					
All locations mean (sd), [°C]	34.4 (0.5)	35.3 (0.4)	34.9 (0.6)	39.6 (1.7)	31.9 (0.8)
Sun locations mean (sd), [°C]	37.9 (1.1)	38.3 (4.5)	39.4 (0.8)	39.6 (1.7)	34.9 (1.3)
Shade locations mean (sd), [°C]	33.0 (0.3)	32.9 (0.3)	31.1 (0.4)	N/A	29.2 (0.8)
Sun-Shade difference, [°C]	4.9	5.4	8.3	N/A	5.7
Values above 1-min skin burn, [%]	0	0	0	0	N/A
Values above 5-s skin burn, [%]	0	0	0	0	N/A
(b) Afternoon					
All locations mean (sd), [°C]	42.2 (0.4)	47.8 (0.6)	51.3 (0.7)	59.8 (0.8)	43.1 (1.2)
Sun locations mean (sd), [°C]	47.7 (1.0)	57.4 (0.5)	60.4 (0.7)	59.8 (0.8)	50.1 (1.6)
Shade locations mean (sd), [°C]	40.4 (2.5)	41.3 (0.5)	44.3 (0.6)	N/A	36.0 (0.7)
Sun-Shade difference, [°C]	7.3	16.1	16.1	N/A	14.1
Values above 1-min skin burn, [%]	7.9	33.6	37.9	83.3	N/A
Values above 5-s skin burn, [%]	0.7	18.3	24.7	44.4	N/A

Independent samples tests revealed that measurements of surface temperature in the sun were significantly different from measurements taken in the shade (*F* = 233.412, *p* < 0.001). Factorial ANCOVA, with local airport ambient temperature as a covariate, for sun and shade exposed materials showed smaller temperature differences between material types in the shade compared to surfaces in the sun (*F* = 27.232, *p* < 0.001, partial eta squared = 0.211 for sun; *F* = 23.41,7 *p* < 0.001, partial eta squared = 0.108 for shade).

**Fig. 3:**
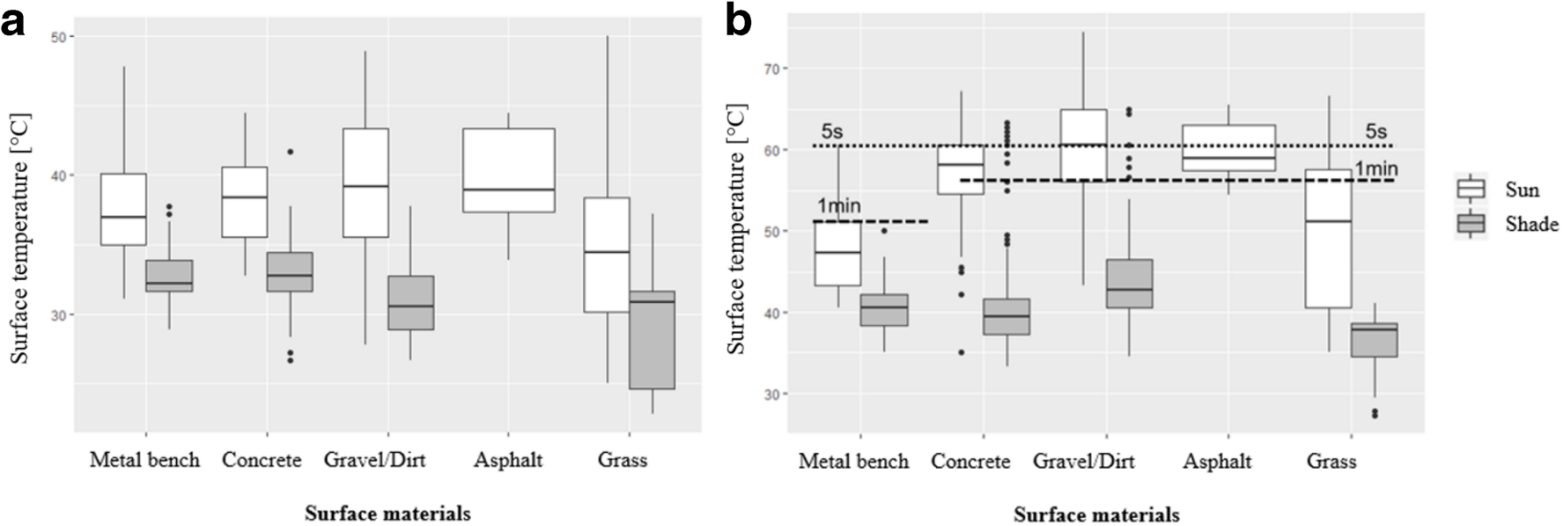
Boxplot of surface temperatures of available materials in the morning (**a**) and afternoon (**b**) in the sun and shade with 5-s and 1-min skin burn threshold collected during the field campaign between June 6 and July 27, 2018 (*N* = 1003)

Surface temperatures of all sampled materials in the morning remained under the 5-s and 1-min exposure skin burn thresholds. However, sun-exposed surfaces for all human-made material types were sufficiently hot to burn human skin at 5-s or 1-min exposure in the afternoon (ISO 13732,2010). No skin burn threshold was available for grass. Shade lowered mean surface temperatures by up to 16 °C in the afternoon (Table 3b) with all surface temperatures for shaded surfaces falling below the skin burn thresholds (Fig. 3b).

### Field surveys

**Fig. 4:**
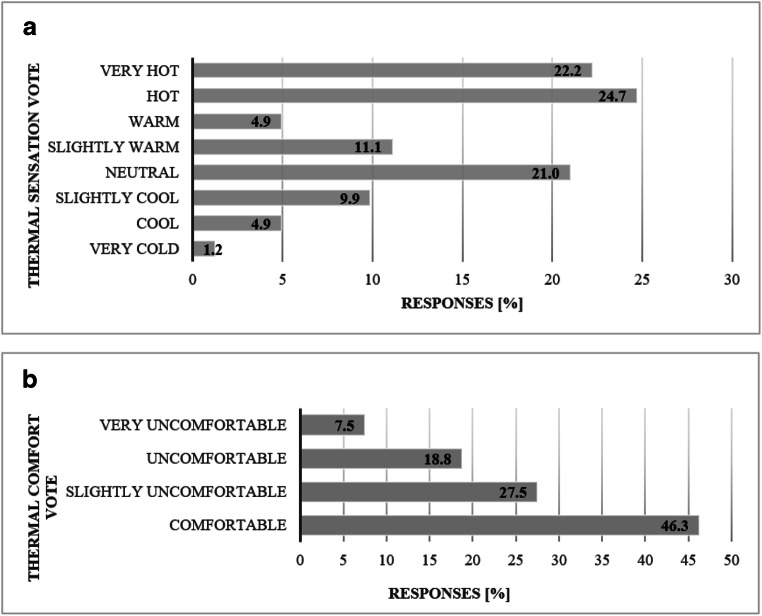
Combined thermal sensation vote (**a**) and combined thermal comfort vote (**b**) for all stop types. No participants voted for “COLD” on a thermal sensation scale. Responses collected during the field campaign between June 6 and July 27, 2018 (*N* = 81)

During the study, we collected 83 questionnaires at six bus stops with a variety of design attributes. A comparison of demographic information we collected on the survey to the regional rider profile reflects disadvantaged conditions of the neighborhood compared to the population of greater Phoenix. In our sample, 52% of the study respondents reported earning less than $20,000 (Table 4) versus 24% for the region. Furthermore, 21% of study respondents owned a vehicle while 32% of public transit system riders in the region had at least one vehicle in their household. Study participants were generally younger than the regional population, with 70% of respondents reporting an age below 35 compared to 49% for the whole region. The respondent pool was slightly skewed toward men compared to the regional transit ridership as a whole (Valley Metro 2019). We did not observe any statistically significant differences in thermal sensation vote or thermal comfort between different demographic groups. Nearly half of study participants felt hot or very hot at the time they were surveyed (Fig. 4a), and 55% experienced some degree of thermal discomfort (Fig. 4b). Thermal comfort was moderately correlated with thermal sensation vote (*r* = 0.495, *p* < 0.001).

**Table 4:** Descriptive statistics of demographic variables collected during the filed campaign between June 6 and July 27, 2018 (*N* = 83)

Demographic variables		**(***N* = 83) [valid%]
Gender	Male	66.7
	Female	33.3
Age	18–25	56.8
	26–35	13.6
	36–50	13.6
	51–65	11.4
	65+	4.5
Income	Below 20,000	52.2
	21,000–30,000	19.6
	31,000–40,000	17.4
	41,000–60,000	4.3
	61,000–80,000	2.2
	81,000–100,000	2.2
	100,000+	2.2
Vehicle ownership	Yes	20.9
	No	79.1
Lived in Phoenix for	Less than 3 months	10.8
	3 months to a year	3.1
	1 to 3 years	9.2
	3+	76.9
Part of the daily routine	Yes	60.9
	No	39.1

**Fig. 5:**
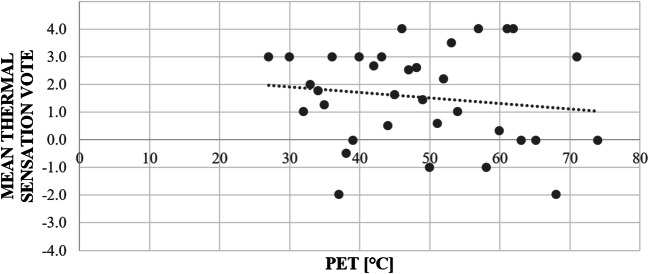
Relationship between mean thermal sensation votes and binned PET; responses collected during the field campaign between June 6 and July 27, 2018 (*N* = 81)

None of the environmental variables we measured was correlated to thermal sensation vote during any time period. To further explore the thermal sensitivity of respondents to environmental conditions, we calculated the mean thermal sensation vote (MTSV) within 1 °C intervals of PET (as in Middel et al. 2016). Linear regression showed no significant relationships between PET and mean thermal sensation votes (*p* = 0.42), and the model had a negative slope (Fig. 5), opposite of the hypothesized direction.

Certain amenities influenced riders’ perception of stop beauty, but not pleasantness. A linear regression model showed that enhanced bus stops with multiple design attributes such as artistic features, trees and shrubs, and vegetated metal trellis were rated as more beautiful compared to standard types of shelters with no or minimal vegetation. There was a half point change on the ugly-to-beautiful scale at stops with improved shelters and vegetation (unstandardized B = 0.541, standardized coefficient beta = 0.26, *p* = 0.019), but relationships for pleasantness were not significant.

Both perception of stop pleasantness and beauty were significantly related to thermal sensation vote. Perception of stop beauty had a stronger influence on thermal sensation vote than did pleasantness. We found that for one unit of change on the ugly-to-beautiful scale, riders felt cooler by 0.8 points on the thermal sensation vote scale (unstandardized B = − 0.80, standardized coefficient beta = − 0.409, *p* < 0.001). For one unit of change from unpleasant to pleasant, riders felt cooler by half a point (unstandardized B = − 0.554, standardized coefficient beta = − 0.314, *p* = 0.004).

**Fig. 6:**
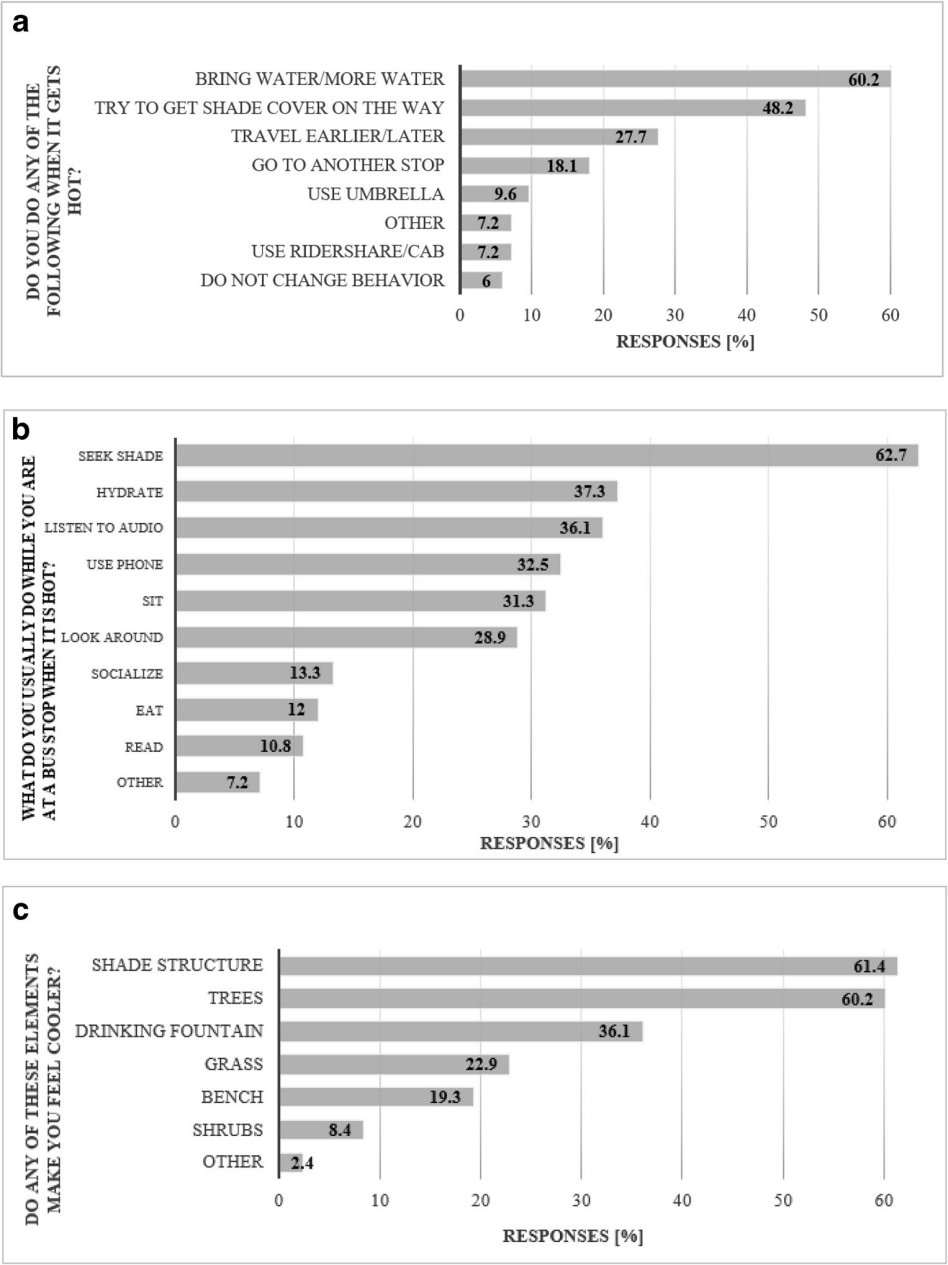
Survey responses to the questions: **a** “Do you do any of the following when it gets hot?”; **b** “What do you usually do while you are at a bus stop when it’s hot?”; **c** “Do any of these elements make you feel cooler?” Multiple-choice options. The figures show the percent of total respondents that selected per each option; respondents could choose more than one option. Responses collected during the field campaign between June 6 and July 27, 2018 (*N* = 83)

Study participants reported a wide variety of strategies to cope with the summer heat during their use of the public transportation system. Searching for shade and hydrating or carrying more water were the predominant coping strategies that survey participants reported while waiting and walking to bus stops (Fig. 6a and b). Shade structures and trees were the infrastructure features identified most often as having perceived cooling benefits. Drinking fountains were perceived as beneficial for cooling by more than a third of respondents (Fig. 6c). Other types of infrastructure that participants self-reported as having cooling benefits included misters, electric plugs, more built shade and seating, water fountains, and natural shade. Even though only 3.6% of riders were under a tree when they took the survey, trees were identified to have cooling benefits by nearly as many participants (60.2%) as were shade structures (61.4%) (Fig. 6c).

## Discussion and conclusions

Many American cities that were designed with personal automobile use in mind are now prioritizing sustainability, mixed-use zoning, and non-motorized and public transit travel. Thermally conscious design in warming climates will help to fulfill sustainable growth and mobility goals for cities where automobile use has been the dominant form of transportation. It can also alleviate heat stress on the most vulnerable population groups, as low-income individuals and minority groups are more likely to be exposed to heat due to higher use of public transit (Karner et al. 2015). We assessed environmental conditions and thermal perceptions at bus stops in a hot and dry US city to understand the magnitude of exposure and thermal perceptions experienced by socioeconomically vulnerable populations. Riders of Hispanic ethnicity represent the largest proportion of transit users among minorities in the Phoenix Metropolitan Area, 23%, and more than a half of Valley Metro public transit system riders earn less than $30,000 annually, underscoring underlying social vulnerabilities of users (West Group Research 2018). Current study was conducted in a predominantly Latinx neighborhood where more than half of survey respondents reported earnings below $20,000 per household, representative of regional patterns.

We found that shade from all sampled design attributes, with the exception of vegetated awnings in the afternoon, significantly reduced mean PET by as much as 21 °C. However, the mean PET we measured in the shade (during all hours) was 45 °C, which is much higher the acceptable thermal comfort threshold of 38.1 °C determined by Middel et al. (2016) for the same hot and dry climate conditions on a university campus. The vegetated awning we measured was only effective in reducing PET in the morning; it failed to provide significant reductions in the afternoon. Many of the vines at the bus stops we sampled were not properly maintained and were dried out. As a result, they had few leaves and did not provide a lot of shade, which decreased their effectiveness in the afternoon. No other statistically significant differences between the shade from different design attributes were detected. Standalone advertising signs near bus stops provided the highest reductions in PET in the morning, emphasizing the need for solid vertical shade integrated into bus stop designs in the study region. In addition, enhanced bus stop type with a polycarbonate canopy was less effective in reducing PET as compared to a standard metal one. This can be explained by the fact that enhanced bus stops did not have vertical panels as well as semi-opaque material of the canopy. Likewise, a study in Malaysia that compared the effectiveness of the opaque bus stop shelter cover with a polycarbonate one, found that PET under the shade from the polycarbonate canopy was consistently higher than under the opaque, and subjective assessment also showed a higher percentage of “comfortable” conditions under the opaque shelter cover (Goshayeshi et al. 2013).

Surface temperatures of all sun-exposed man-made materials exceeded skin burn thresholds in the afternoon. This finding was particularly concerning, because people experiencing homelessness, mental illness, and substance abuse, or simply in need of respite, may sit on pavement or bus stop benches for extended periods and are at risk for skin burns. The dangerously hot surfaces we found also pose high risks to children, who have more sensitive skin than adults and are thus more susceptible to burn injuries from touching hot surfaces (Vanos et al. 2016). Even though grass was the coolest material we sampled on average, sun-exposed grass surface temperatures were very high, with maximums above 66 °C. No skin burn threshold was available for grass; however, we suspect that exposure to such temperatures could also be dangerous. Shade was highly effective in lowering the surface temperature below burn thresholds across all surfaces we examined, similar to findings reported for playground surfaces in the same climatic setting (Vanos et al. 2016).

Trees and bus shelters were highly valued by the bus riders we surveyed. Bus stops with more design attributes were rated as more beautiful, even though no improvements in microclimate were observed. Moreover, riders with higher perceptions of beauty and pleasantness reported lower thermal sensation. This finding is likely related to the psychological aspects of thermal comfort, such as perceived control of the environment (Nikolopoulou and Steemers 2003) and importance of pleasantness in design, as well as alliesthesia through perceiving more shade options as beneficial for cooling (Heng and Chow 2019; Johansson et al. 2018). Furthermore, standard bus stops did not provide any combined effect of gray and green infrastructure in reducing ambient and mean radiant temperature due to a lack of the latter. The absence of green infrastructure could have a negative effect on psychological aspects of thermal comfort, because of limited perceived control of the environment and a perceived lack of naturalness (Nikolopoulou and Steemers 2003).

Infrastructure improvements suggested by the study participants, such as misters, electric plugs, more built shade, seating, and water fountains have the potential to improve both physiological and psychological aspects of thermal comfort. While misters, additional vegetation, shade, and water fountains have apparent cooling benefits, electric plugs, Wi-Fi, or real-time bus arrival information at stops could reduce perceived wait time (Fan et al. 2016; Watkins et al. 2011) and potentially alleviate psychological thermal discomfort. Access to shade and drinking water was selected as the most preferred heat-coping strategy. Respondents’ preferred coping strategies further emphasize the importance of the psychological aspects of thermal comfort. For instance, many people selected trees as important cooling elements even though they were not using their direct shade at the time they were surveyed.

The high PET values we observed in this study generally corresponded with respondents’ perceptions of the thermal environment. Almost half of the riders we surveyed reported feeling hot or very hot, and more than a half experienced some degree of thermal discomfort. We expect that this response invariance explains why PET and microclimate variables were not statistically significant predictors of thermal sensation vote and thermal comfort in this study. In addition, at PET above 54 °C, the range of MTSV increased, varying between cool and very hot. Similar results have been reported elsewhere: Middel et al. (2016) showed that at PET above 55 °C there was wide variability of MTSV ranging from slightly warm to very hot for same hot and dry climate conditions. Low MTSVs at high PET values could be possibly explained by the psychological mechanisms of avoidance coping (Krohne 2001) with extreme heat. Other studies in areas with similar climate show that at PET between 37 and 50 °C, relationships between MTSV and PET tend to flatten (Cohen et al. 2019). Since in this study all PET values were high, with only 18% of measurements below 37 °C, it is possible that participants’ sensitivity to further PET increases was minimal.

This research has several limitations related to the process of data collection and the infrastructure and demographic conditions in the region. First, we expect that our results related to thermal conditions at bus stops, and riders’ experiences, are conservative because we only sampled at north-facing bus stops. Stops facing other directions are likely to have even more adverse conditions because they are less protected from sunlight. Our modest survey sample size reflected low public transit use in Phoenix (3%), where the car is by far the predominant commuting mode (87%) (Phoenix, AZ | Data USA 2016). Since participants were asked to fill out the survey while waiting for the bus, riders who came to the stop less than 5 min before the bus arrival did not always have time to complete the survey. Thus, demographic questions that were at the end of the survey were often incomplete, or some people could not participate entirely due to lack of time. Another consideration is that Tmrt derived from the globe temperature measured with Kestrel 4400 Heat Stress Meter is likely to be overestimated. When this sensor’s small black powder-coated globe is exposed to the sun, it absorbs too much short wave radiation and it also has a longer response time compared to the standard black globe thermometer (Kántor and Unger 2011; Middel et al. 2016). In addition, a smaller globe is more sensitive to wind variations, which can lead to up to 0.5 °C differences in globe temperature (Johansson et al. 2018). This overestimate may lead to the PET values reported in this study being slightly to moderately higher than we would have estimating using other instrumentation in the field.

Overall, strategic placement of green and gray infrastructure elements to provide shade throughout the day, and most importantly in the afternoon, and careful consideration of material properties with high albedo and lower heat conductance, can help to decrease thermal exposure (Vanos et al. 2016). Improving aesthetic perceptions of bus stop infrastructure and diversifying design attributes at bus stops have the potential to improve thermal comfort of bus riders through improved perception of beauty and pleasantness. In addition, guidelines for design collaborations with artists should include conditions to use materials and structures that prioritize shade in thermally challenged climates. For instance, PET at bus stops with integrated artistic features could potentially be improved if opaque shade cover was used and vine trellis structures were properly maintained or augmented with additional shading, such as fabric sails, or vertical shade panels that are currently absent at those types of stops. Thus, thermally conscious design needs to be a priority in cities challenged by climate extremes, especially because of the coupling of public transportation systems and cities’ larger goals for sustainability and well-being.

## Supplementary information

ESM 1 (PDF 451 kb)

## Data Availability

Data are available online: https://portal.edirepository.org/nis/mapbrowse?scope=edi&identifier=506&revision=1

## Footnotes

1 High standard deviation is a result of significantly higher daily average ridership at several rapid transit bus stops in downtown core, comparing to the rest of the city.
