# Supplementary material for: Public transit infrastructure and heat perceptions in hot and dry climates
Source: Int J Biometeorol. 2021 Jan 26;66(2):345–56. doi: 10.1007/s00484-021-02074-4 (PMC8807448; doi:10.1007/s00484-021-02074-4)
Supplement: Supplementary file 1 — (PDF 451 kb) [file 484_2021_2074_MOESM1_ESM.pdf]

Public Transit Infrastructure and Heat Perceptions in Hot and Dry Climates

Y. Dzyuban ● D. M. Hondula ● P. J. Coseo ● C. L. Redman

SUPPLEMENTARY MATERIAL

Survey instrument used during the field campaign between June 6 and July 27, 2018

To be filled out by survey administrator
Team member name: __________
Bus stop ID#: ________________

ARIZONA STATE UNIVERSITY

City of Phoenix Bus Stop Survey, Summer 2018

ASU research team requests your help in understanding public transit riders' experiences at Phoenix bus stops. Please complete the following questions to the best of your ability. All answers are optional. Thank you for your time.

1. How did you reach this bus stop?

☐ Walking ☐ Biking ☐ In a vehicle ☐ Transferred from another bus ☐ Other: ______

2. How long did it take you to reach this bus stop?

☐ 1-5 minutes ☐ 6-10 minutes ☐ 11-15 minutes ☐ 16-20 minutes ☐ Over 20 minutes

3. How long do you typically wait at this bus stop?

☐ 1-5 minutes ☐ 6-10 minutes ☐ 11-15 minutes ☐ 16-20 minutes ☐ Over 20 minutes

4. Do you do any of the following when it gets hot? Select all that apply.

☐ Earlier/later travel ☐ Bring an umbrella ☐ Bring water or bring more water

☐ Rideshare/cab ☐ I try to get shade cover on the way ☐ Go to another stop with more shade

☐ I don't change my behavior ☐ Other: ______________

5. What do you usually do while you are at a bus stop when it's hot? Select all that apply.

☐ Seek shade ☐ Sit ☐ Look around ☐ Use phone ☐ Hydrate ☐ Eat ☐ Socialize

☐ Listen to audio ☐ Read ☐ Other: ________

6. Do any of these elements make you feel cooler? Select all that apply.

☐ Nearby trees ☐ Nearby grass ☐ Nearby shrubs ☐ Benches ☐ Shade Structures

☐ Nearby drinking water fountain ☐ Other: ________________

7. What is your perception of this bus stop?

☐ Unpleasant ☐ Slightly unpleasant ☐ Neutral ☐ Slightly Pleasant ☐ Pleasant

8. Do you think this bus stop is:

☐ Ugly ☐ Somewhat ugly ☐ Neutral ☐ Somewhat beautiful ☐ Beautiful

9. How do you feel at this bus stop?

☐	☐	☐	☐	☐	☐	☐	☐	☐
Very Cold	Cold	Cool	Slightly Cool	Neutral	Slightly Warm	Warm	Hot	Very Hot

10. My current level of thermal comfort is:

☐ Comfortable ☐ Slightly Uncomfortable ☐ Uncomfortable ☐ Very Uncomfortable

Personal background:

Using this bus stop is part of my daily routine: ☐ Yes ☐ No

I have lived in Phoenix for: ☐ Less than 3 months ☐ 3 months to 1 year ☐ 1-3 years ☐ 3+ years

Do you own a vehicle? ☐ Yes ☐ No

Reason for trip: ☐ Work/School ☐ Family/friends ☐ Recreation ☐ Errands/shopping

☐ Other: ___________

Household income:

☐ Below $20,000 ☐ $21,000-$30,000 ☐ $31,000-$40,000 ☐ $41,000-$60,000

☐ $61,000-80,000 ☐ $81,000-$100,000 ☐ Over $100,000

Age: ☐ 18-25 ☐ 26-35 ☐ 36-50 ☐ 51-65 ☐ 65+

Is there anything else you'd like to tell us about bus stops in Phoenix?

Thank you very much for taking the time to complete this survey. Your feedback is valued and very much appreciated!
